# Anti-Inflammatory Effect of Resveratrol Derivatives via the Downregulation of Oxidative-Stress-Dependent and c-Src Transactivation EGFR Pathways on Rat Mesangial Cells

**DOI:** 10.3390/antiox11050835

**Published:** 2022-04-25

**Authors:** I-Ta Lee, Horng-Chyuan Lin, Tse-Hung Huang, Chi-Nan Tseng, Hao-Tsa Cheng, Wen-Chung Huang, Ching-Yi Cheng

**Affiliations:** 1 School of Dentistry, College of Oral Medicine, Taipei Medical University, Taipei 11031, Taiwan; itlee0128@tmu.edu.tw; 2 Department of Thoracic Medicine, Chang Gung Memorial Hospital at Linkou, Taoyuan 33305, Taiwan; College of Medicine, Chang Gung University, Taoyuan 33302, Taiwan; lhc53424@cgmh.org.tw; 3 Graduate Institute of Health Industry Technology, Research Center for Chinese Herbal Medicine and Research Center for Food and Cosmetic Safety, Chang Gung University of Science and Technology, Taoyuan 33303, Taiwan; kchuang@cgmh.org.tw (T.-H.H.); wchuang@mail.cgust.edu.tw (W.-C.H.); 4 Department of Traditional Chinese Medicine, Chang Gung Memorial Hospital, Keelung 20401, Taiwan; 5 School of Nursing, National Taipei University of Nursing and Health Sciences, Taipei 11219, Taiwan; 6 Division of Cardiac Surgery, Department of Thoracic and Cardiovascular Surgery, Chang Gung Memorial Hospital at Linkou, Taoyuan 33305, Taiwan; chinan.tseng@cgmh.org.tw; 7 Department of Molecular Medicine and Surgery, Karolinska Institute, 17177 Stockholm, Sweden; 8 Department of Medicine, Chang Gung University, Taoyuan 33302, Taiwan; hautai@adm.cgmh.org.tw; 9 Department of Gastroenterology and Hepatology, Chang Gung Memorial Hospital at Linkou, Taoyuan 33305, Taiwan; 10 Graduate Institute of Clinical Medicine, College of Medicine, Chang Gung University, Taoyuan 33302, Taiwan; 11 Division of Allergy, Asthma and Rheumatology, Department of Pediatrics, Chang Gung Memorial Hospital at Linkou, Taoyuan 33305, Taiwan; 12 Department of Pediatrics, New Taipei Municipal TuCheng Hospital, New Taipei 23652, Taiwan; Chang Gung Memorial Hospital at Linkou, Taoyuan 33305, Taiwan; 13 Department of Pulmonary Infection and Immunology, Chang Gung Memorial Hospital at Linkou, Taoyuan 33305, Taiwan

**Keywords:** Res derivatives, anti-oxidation, anti-inflammation, transactivation EGFR

## Abstract

In Taiwan, the root extract of *Vitis thunbergii* Sieb. et Zucc. (Vitaceae, VT) is rich in stilbenes, with resveratrol (Res) and its derivatives being the most abundant. Previously, we showed that the effect of Res derivatives against tumor necrosis factor-α (TNF-α)-stimulated inflammatory responses occurs via cPLA2/COX-2/PGE2 inhibition. This study compared and explored the underlying anti-inflammatory pharmacological mechanisms. Before stimulation with TNF-α, RMCs were treated with/without pharmacological inhibitors of specific protein kinases. The expression of inflammatory mediators was determined by Western blotting, gelatin zymography, real-time PCR, and luciferase assay. Cellular and mitochondrial ROS were measured by H_2_DHFDA or DHE and MitoSOX™ Red staining, respectively. The RNS level was indirectly measured by Griess reagent assay. Kinase activation and association were assayed by immunoprecipitation followed by Western blotting. TNF-α binding to TNFR recruited Rac1 and p47^phox^, thus activating the NAPDH oxidase-dependent MAPK and NF-κB pathways. The TNF-α-induced NF-κB activation via c-Src-driven ROS was independent from the EGFR signaling pathway. The anti-inflammatory effects of Res derivatives occurred via the inhibition of ROS derived from mitochondria and NADPH oxidase; RNS derived from iNOS; and the activation of the ERK1/2, JNK1/2, and NF-κB pathways. Overall, this study provides an understanding of the various activities of Res derivatives and their pharmacological mechanisms. In the future, the application of the active molecules of VT to health foods and medicine in Taiwan may increase.

## 1. Introduction

Mesangial cells (MCs) are mostly found in the glomerular stalk, where they structurally support glomerular capillaries. They are stromal cells and share similarities with fibroblasts, pericytes, and smooth muscle cells. Therefore, they may help the glomerular vasculature respond to various physical stimuli. MCs play a major role in glomerular development, phagocytosis, and glomerular basement membrane matrix production. In addition, they participate in the evolution of immune-mediated renal inflammatory diseases, such as glomerulonephritides (GN), glomerulosclerosis, and glomerular hypercellularity [1], since MCs under certain conditions produce inflammatory mediators, such as tumor necrosis factor (TNF)-α [2].

The main cause of acute kidney injury is the clinical oxidative pressure damage to the kidney caused by ischemia and reperfusion [3]. Oxidative stress is an imbalance between the production of free radicals and the ability of antioxidants, which causes oxidative damage and change kidney tissue. Free radicals include reactive free radicals and non-radical derivatives of reactive oxygen species (ROS) and reactive nitrogen species (RNS). The latter is considered critical in the pathogenesis and progression of chronic kidney disease (CKD) [4,5]. High-oxygen-consuming organelles, such as mitochondria, are the main source of endogenous RONS. In addition, NADPH oxidase (Nox) is a common source of superoxide free radicals (^•^O_2_^−^) in cells. Most superoxides interact with peroxides (H_2_O_2_) produced by superoxide dismutase (SOD) and then, through Fenton or Haber–Weiss reactions, generate free radicals (^•^OH), thus damaging cells. There are three sources of nitric oxide (NO) in mammals. Among them, inducible nitric oxide synthase (iNOS) can be activated by the stimulation of different endotoxins or cytokines and uses arginine and oxygen to produce NO. NO then reacts with free radicals (^•^O_2_^−^) to form peroxynitrite (ONOO^−^, RNS), thus damaging cells.

Gelatinases, including MMP-2 and MMP-9, are members of the MMP family; they play an important role in the degradation of the extracellular matrix. Therefore, they are also critical in the development and progression of CKD. Cell signaling pathways, hypoxia, and changes in cell membrane structure all affect their expression and activity. Gelatinases can also affect the development of CKD by interacting with TNFs, monocyte chemoattractant proteins, growth factors, oxidative stress, etc. In the early stage of CKD, when the kidney is damaged, damaged cells and inflammatory cells secrete a variety of pro-inflammatory and pro-fibrotic cytokines to promote renal interstitial fibrosis. Concomitantly, the activities of MMP-2 and MMP-9 increase, which damages the kidney’s basement membrane, promotes the phenotypic transformation of renal tubular epithelial cells, and ultimately leads to the aggravation of ECM deposition. However, in the late stage of CKD, the activities of MMP-2 and MMP-9 are reduced, resulting in insufficient ECM degradation; therefore, fibrosis is difficult to reverse. The decrease of MMP-2 activity in the late stage of CKD is related to the increased endocytosis caused by hypoxia. However, the mechanism underlying the decline of MMP-9 activity remains unclear [6].

*Vitis thunbergii* Sieb. et Zucc. (Vitaceae, VT) root extracts contain many resveratrol (Res) oligomers, such as vitisinols A–D, (+)-ε-viniferin, (−)-viniferal, ampelopsin C (AC), miyabenol A, (+)-vitisin A, and (+)-vitisin C. In addition to vitisinol A, other Res oligomers have good antioxidant and anti-platelet activation capabilities [7,8]. The root of VT contains many Res oligomers and polyphenol compounds, all of which are stilbenes. Therefore, VT extracts or purified compounds have anti-inflammatory, anti-hypertensive, and cardiac and neuroprotective effects [9,10,11,12]. Res is a natural plant polyphenol with many biological activities, such as antioxidant, anti-inflammatory, anti-cancer, anti-diabetic, cardioprotective, and even anti-aging properties [13,14,15,16,17,18]. Recently, many studies have demonstrated that Res oligomers are more effective in inhibiting tumor cell proliferation, inflammation, and antifungal activity compared with Res [19,20,21]. AC is a Res trimer that has an excellent anti-inflammatory effect [11], (+)-vitisin A and AF are Res dimers [22], and polydatin (PD) is a trans-Res glucoside with proven significant anti-platelet and antioxidant activity [7,20] and anti-inflammatory biological activity [23,24]. However, no articles in the literature have discussed VT’s effect on renal disease in detail. Therefore, this study first evaluated and compared the anti-inflammatory effects of the active molecules of VT on rat renal mesangial cells (RMCs) and explored their underlying pharmacological mechanisms.

## 2. Materials and Methods

### 2.1. Materials

Professor Chuan-fu Lin from the Department of Cosmetics at Chang Gung University of Science and Technology and Professor Yu-ling Huang from the National Institute of Chinese Medicine provided VT purified products from Taiwan (AC, AF) [7,8]. The methods of extraction and purification of these compounds have been clearly explained in previous studies [11]. Res, PD, GAPDH, and other antibodies, enzymes, inhibitors, chemical reagents, or powders were purchased from Merck-Millipore, Darmstadt, Germany. The DMEM culture medium was purchased from Gibco BRL, FBS was from HyClone, and TRIzol was from Invitrogen. Polyvinylidene fluoride (PVDF) membranes were from Millipore. The enhanced chemiluminescence (ECL) kit was purchased from Visual Protein Biotech. TNF-α was from R&D Systems. The purified VT, Res, and PD were all dissolved in dimethyl sulfoxide (DMSO). The concentrations of the products purified from the VT, Res, and PD that were required for each cell experiment were determined, and the reagents were diluted to the final concentration with a serum-free cell culture medium. The control group for each cell experiment analysis was 1% DMSO.

### 2.2. Cell Culture

The cell culture method used here was that described in previously published articles [11,25]. When the number of cells exceeded about 80% of the Petri dish area, the RMCs (from the American Type Culture Collection (ATCC; Rockville, MD, USA)) were replaced with a serum-free culture medium and cultured for 1 day before the biochemical analyses. AC, AF, PD, and Res were added for 2 h first, followed by stimulation with TNF-α (10 ng/mL) at different times according to the various experiments.

### 2.3. Protein Concentration Measurement

After the treatment of cells with the drug, a RIPA lysis buffer (50 mM Tris-HCl, pH 7.5, 150 mM NaCl, 0.5% sodium deoxycholate, 1% IGEPAL^®^ CA-630, and 0.1% SDS) with protease inhibitor and phosphatase inhibitor cocktails (Merck-Millipore, Darmstadt, Germany) was used to break the cell membrane. The concentration of the cell lysate was determined using the Pierce bicinchoninic acid (BCA) protein assay kit (Thermo Fisher Scientific, Rockford, IL, USA). According to the operation steps described in the manufacturer’s manual, the absorbance value was measured at a wavelength of 562 nm. The protein concentration of the sample was calculated according to the calibration curve of the standard.

### 2.4. Detection of Free Radicals in Cells

When the number of cells in the starvation state was about 90%, H_2_DCFDA (4 μM) was added at 37 °C for 30 min. After washing with PBS, the cells were incubated with or without AC, AF, Res, and PD for 2 h and then stimulated with TNF-α (10 ng/mL) for 6 h or different times. The cells were lysed with lysis buffer and centrifuged at 10,000 rpm at 4 °C for 10 min. The H_2_DCF fluorescence value (H_2_O_2_ detection) of the supernatant was read on a SpectraMax i3 microplate reader (Molecular Devices, San Jose, CA, USA) (excitation at 485 nm, emission at 530 nm). Moreover, the BCA kit was used to measure protein concentrations. The free radical generation rate was determined as follows: fluorescence value of the analyte/protein concentration of the analyte)/(fluorescence value of the control group/protein concentration of the control group). In addition, after treatment with the drug, the cells (1 × 10^6^ cells/mL) were collected and stained with dihydroethidium (DHE) in the Muse^®^ Oxidative Stress Kit to detect ^•^O_2_^−^, and a Muse^®^ Cell Analyzer (Merck-Millipore, Darmstadt, Germany) was used for analysis. To detect the free radicals generated by the respiratory electron transport chain in the mitochondria, we planted cells on glass slides, fixed them, used MitoSOX™ Red fluorescent markers to quantify the free radicals, and finally observed them using an upright fluorescence microscope (DFC310FX; Leica, Wetzlar, Germany). The distribution of free radicals in the cells was analyzed via ImageJ software-based quantitative analysis (NIH). For each treatment, six different fields of view were assessed for quantification.

### 2.5. DPPH^•^ Free Radical Scavenging Analysis

Freshly prepared DPPH^•^ (200 µM) was reacted with AC, AF, Res, and PD in serial dilutions for 20 min in a dark room at 25 °C and then detected by measuring the absorbance at 517 nm.

### 2.6. NO Content Analysis

The method used for the analysis of NO content was that described in a previously published article [25]. Briefly, 50 μL of Griess reagent containing 4% sulfanilic acid, 0.2% *N*-(1-naphthyl) ethylenediamine dihydrochloride, and 10% phosphoric acid were reacted with an equal volume of the cell supernatant after treatment with the drug. After reaction for 10 min at 25 °C in the dark, the absorbance was read at 550 nm. A standard concentration curve of NaNO_2_ was used to calculate the amount of NO produced by the sample.

### 2.7. Western Blotting

The method used for Western blotting was that described in previously published articles [11,25]. The samples were loaded into 10% SDS–PAGE and, after electrophoresis and transfer, the PVDF membrane was reacted with primary and secondary antibodies, followed by reaction with the ECL reagent. Luminescence images were captured and quantified using the ChemiDoc XRS+ system and the Image Lab^TM^ 6.0.1 Software (Bio-Rad Laboratories, Inc., Hercules, CA, USA). The target protein expression was calculated as follows: target protein quantitative value/GAPDH quantitative value; then, the ratio was compared with the control group and presented in multiples.

### 2.8. Gelatin Zymography

The method used for gelatin zymography was that described in a previously published article [25]. After treating the cells with the drugs, the culture medium was collected and centrifuged at 4 °C at 10,000 rpm for 10 min to remove cell debris. The supernatant was collected, mixed with 5× non-reduced sample buffer, and loaded into the sample wells of SDS–PAGE containing 1% gelatin (as the substrate of MMP-2 or MMP-9). After the completion of electrophoresis, the gel was washed with 3% Triton X-100 for about 30 min to remove SDS and then replaced with a developing buffer (50 mM Tris, 40 mM HCl, 200 mM NaCl, 5 mM CaCl_2_, and 0.2% Brij 35) at 37 °C for 2–7 days. Finally, the gel was stained with stain buffer (30% methanol, 10% acetic acid, and 0.5% (*w*/*v*) Coomassie Brilliant Blue). A transparent band was detected at the molecular weight of MMP-2 and MMP-9, representing the enzymatic activity of MMP-2 and MMP-9 on the blue gel. ImageJ software was used to quantify the zona pellucida.

### 2.9. Reverse Transcription and Real-Time PCR

The method used to carry out these procedures was that described in previously published articles [11,25]. After treatment with the drug, RNA was extracted with the TRIzol reagent (Invitrogen, Carlsbad, CA, USA). Using RNA as a template, reverse transcription into cDNA was performed using the first-strand cDNA synthesis kit (Bio-Rad). The SoFast EvaGreen Supermix (Bio-Rad) and the target primers were mixed evenly with 50 ng of cDNA in the test tube and placed in a CFX Connect Real-time PCR Detection System machine (Bio-Rad) for reaction. The cycling conditions were as follows: 40 cycles of 3 min at 95 °C and 10 s at 95 °C, followed by 30 s at 58 °C. Each group of samples had three replicates and an endogenous control group (β-actin). The expression levels of genes were calculated in the form of 2^–ΔΔCt^, with Ct referring to the threshold cycle. The expression level of each gene was first standardized with β-actin and then divided by the value of the control group and presented as a relative multiple. The primers used for real-time PCR were as follows: MMP-9 (accession number: NM_031055) sense: 5′–CGC CAG CCG ACT TAT GTC–3′, anti-sense: 5′–CAG GTA ATC CTC TGC CAG C–3′; COX-2 (accession number: NM_017232) sense: 5′–CAA GAA TCA AAT TAC CGC TGA AG–3′, anti-sense: 5′–CGA AGG AAG GGA ATG TTG TT–3′; and β-actin (accession number: NM_031144) sense: 5′–CGT GAA AAG ATG ACC CAG ATC A–3′, anti-sense: 5′–CTC CGG AGT CCA TCA CAA TG–3′.

### 2.10. Electroporation and Luciferase Assay

The methods used for electroporation and luciferase assay were those described in a previously published article [11]. The rat MMP-9 (accession number: AJ438266) or COX-2 (accession number: L11611) promoter region was designed to be cloned into the pLightSwitch_Prom vector (SwitchGear Genomics, Inc, Carlsbad, CA, USA), which contains the luciferase reporter system. These sequences were inserted between the MluI and BglII restriction sites. The EasyPrep EndoFree Maxi Plasmid Extraction Kit (Biotools Co., Ltd., New Taipei City, Taiwan) was used to prepare plasmids and for their extraction, purification, and collection according to the instructions of the manufacturer. Subsequently, 1 × 10^6^ RMCs/mL were placed in a 2-mm electroporation cuvette (Bio-Rad) containing 400 μL of Opti-MEM medium and 2.5 µg of MMP-9– or COX-2–luciferase reporter plasmids. Next, the electroporation cuvette was placed in the Gene in the Pulser Xcell^TM^ Electroporation System (Bio-Rad) instrument slot, and a high-voltage electric pulse was applied (260 V, 950 μF, ∞ Ω, exponential decay pulse). The electrified cells were seeded in a 6-well plate culture dish, and antibiotic-free 10% FBS medium was added, followed by culture for 24 h. After treatment with the drug, the cell lysate was collected and processed according to the operation steps of the luciferase assay system (Promega, Madison, WI, USA). Finally, luminescence was measured on a SpectraMax i3 microplate reader (Molecular Devices). Luciferase activity was recorded as the value of luciferase normalized to the value of β-galactosidase. The ratio of the sample divided by the control was presented in relative multiples.

### 2.11. Immunofluorescence Staining

Cells were pre-treated with or without inhibitors for 1 h and then cultured with TNF-α (10 ng/mL) for 5 min. After the reaction, the cells were washed with PBS, then fixed with 4% paraformaldehyde. Moreover, 0.1% Triton X-100 was used to increase cell permeability, and blocking buffer (Visual Protein Biotech Corporation, Taipei, Taiwan) was used for non-specific antigen shielding. Finally, cells were stained with anti-phospho-NF-κB primary and goat anti-rabbit GFP secondary antibodies, followed by observation of the NF-κB distribution in the cytoplasm and nucleus using a fluorescence microscope (DFC310FX; Leica, Wetzlar, Germany). DAPI (a blue-fluorescent DNA dye that stains the nucleus) was also used. For detailed steps, please refer to the previously published article [11].

### 2.12. Co-Immunoprecipitation

The concentration of the whole-cell lysate (the input) was adjusted to 1 mg and reacted with 2 µg of an anti-TNFR1 or anti-EGFR antibody at 4 °C for 24 h. After the reaction, 10 µL of 50% protein A-agarose magnetic beads were added and mixed evenly at 4 °C for 1 day. Magnetic force was used to collect the antigen–antibody conjugates, which were then washed three times with lysis buffer (without Triton X-100), followed by their preparation as the electrophoresis sample in 5× Laemmli buffer and processing through Western blotting with an anti-TNFR1, anti-TRAF2, anti-Rac1, anti-p47, anti-P-c-Src, anti-EGFR, or anti-P-EGFR primary antibody.

### 2.13. Statistical Analysis

The results presented here were calculated and statistically analyzed using the GraphPad Prism Program 7.0 software (Graph Pad, San Diego, CA, USA). The results are expressed as the mean ± standard deviation (SD). Nonparametric ordinary one-way analysis of variance (ANOVA) was used for multiple comparisons, and Dunnett’s post hoc analysis was used to correct for multiple comparisons. Statistical mixed comparisons among groups were performed using two-way ANOVA followed by Tukey’s multiple comparisons test post hoc analysis. Significance was set at *p* < 0.05.

## 3. Results

### 3.1. Res and Its Derivatives Attenuate TNF-α-Induced MMP-9 Expression in RMCs

**Figure 1:**
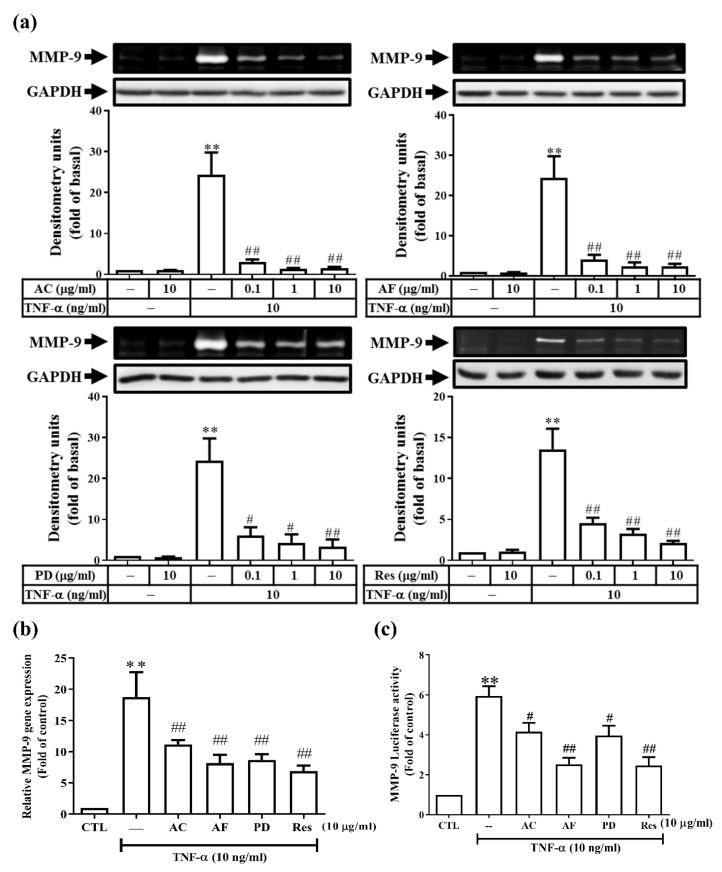
Res and its derivatives attenuated TNF-α-induced MMP-9 expression in RMCs. (**a**,**b**) Cells were treated with different concentrations of AC, AF, PD, or Res for 2 h, followed by stimulation with TNF-α for 48 h (**a**) or 24 h (**b**). (**a**) MMP-9 enzyme activity was determined by gelatin zymography, and Western blotting analyses were used to determine the expression of GAPDH (as a loading control). (**b**) The MMP-9 mRNA transcripts were determined by real-time PCR. (**c**) Cells were transformed with the MMP-9 promoter-luciferase plasmids by electroporation. After the same treatment, a luciferase assay was used to analyze the promoter activity of MMP-9. The results are presented as the mean ± standard deviation (SD) from 4–6 experiments and analyzed using one-way ANOVA followed by Dunnett’s post hoc test. ** *p* < 0.01, compared with the untreated group. ^#^ *p* < 0.05, ^##^ *p* < 0.01, compared with the TNF-α treatment group.

The expression of gelatinases at different stages of CKD is critical for the development and progression of the disease. In addition, the anti-inflammatory effect of Res and its derivatives extracted from VT roots has been reported [11]. Therefore, we first evaluated and compared the effect of these active molecules on the expression of gelatinases in rat RMCs. TNF-α was used to induce an inflammatory model in rat RMCs. We showed that TNF-α induced pro-MMP-9 protein expression in a time- and dose-dependent manner. However, there was no effect on the expression of pro-MMP-2 or on the activities of the two enzymes (Supplementary Figure S2a). In addition, TNF-α upregulated the MMP-9 mRNA transcripts and promoter activity in a time-dependent fashion (Supplementary Figure S2b,c). All of these active molecules abolished the pro-MMP-9 protein expression induced by TNF-α (Figure 1a). We further examined whether the protein inhibition was reflected at the RNA level. As shown in Figure 1b,c, all of these active molecules attenuated both the TNF-α-induced pro-MMP-9 mRNA level and promoter activity. Taken together, these results indicate that Res and its derivatives alleviate the TNF-α-induced pro-MMP-9 expression in RMCs.

### 3.2. Ability of Res and Its Derivatives to Scavenge Free Radicals

**Figure 2:**
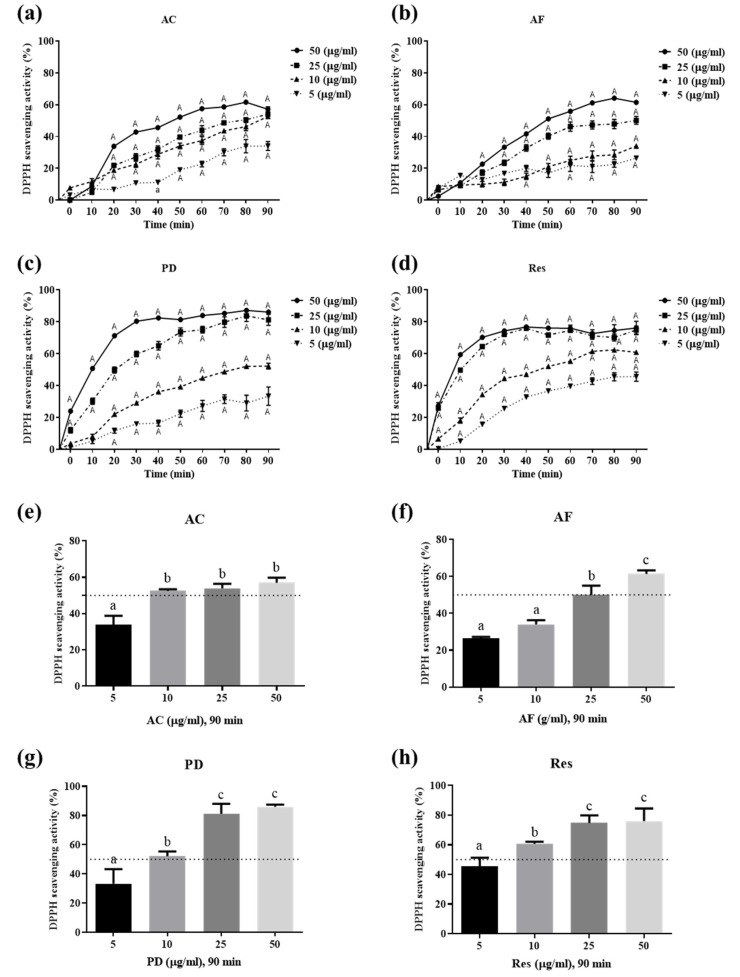
Effects of AC, AF, Res, and PD on the scavenging ability of DPPH^•^ free radicals. (**a**,**e**) AC; (**b**,**f**) AF; (**c**,**g**) PD; and (**d**,**h**) Res reacted with DPPH^•^ radicals for various times, and a SpectraMax i3 microplate reader detected the absorbance values at 520 nm. The results are presented as the mean ± SD from 4 experiments and analyzed (**a**–**d**) using two-way ANOVA followed by Tukey’s multiple comparisons post hoc test. ^a^ *p* < 0.05, ^A^ *p* < 0.01, compared with the untreated group at the same concentration of the drugs; (**e**–**h**) using one-way ANOVA followed by Tukey’s multiple comparisons post hoc test. *p* < 0.05, and different letters represent significant differences between groups.

To determine whether these active molecules scavenge free radicals, the 2,2-diphenylpicrylhydrazyl free radical (DPPH^•^) was used to interact with these active molecules at various time points. Among them, PD and Res at 50 μg/mL had similar clearance rates. Moreover, the free radical scavenging rate of PD and Res at a concentration >25 μg/mL could reach about 80% within 30 min (Figure 2c,d). The clearance rate of PD at 10 μg/mL could reach 50% within 90 min, whereas that of Res at the same concentration could reach 50% within only 50 min (Figure 2c,d). However, the scavenging of DPPH^•^ free radicals by AC and AF at 50 μg/mL was slow, with the scavenging rate not exceeding 50% until 50 min. Furthermore, 80 min were required to reach a clearance rate of only about 60% maxima (Figure 2a,b). The comparison of the IC_50_ of the scavenging of DPPH^•^ free radicals within 90 min yielded the following results: AC and PD, about 10 μg/mL; AF, about 25 μg/mL; and Res, about 5 μg/mL (Figure 2e–h). Res had the best DPPH^•^ clearance rate compared with Res oligomers and PD within 90 min (Figure 2). Taken together, these findings suggest that Res is the best active molecule in the VT root extract for scavenging free radicals in terms of structure.

### 3.3. Res and Its Derivatives Relieve TNF-α-Induced Oxidative Stress in RMCs

**Figure 3:**
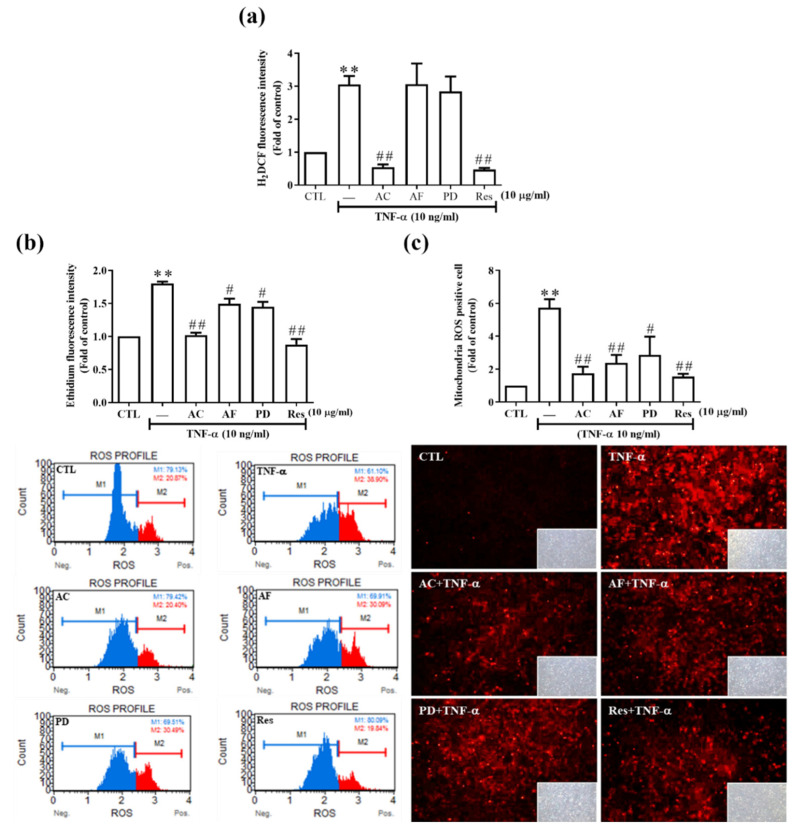
Antioxidant ability of AC, AF, Res, and PD in cells. (**a**) H_2_DCFDA (4 µM) was added to starved cells at 37 °C for 30 min, and cells were pre-treated with Res, AC, AF, and PD (10 μg/mL), cultured for 2 h, and then stimulated with TNF-α (10 ng/mL) for 6 h. Cell lysates were collected and the H_2_DCF fluorescence values were read on a SpectraMax i3 microplate reader (excitation at 485 nm, emission detection at 530 nm); *n* = 4–8. (**b**) After the same treatment as that described in (**a**), cells were harvested (1 × 10^6^ cells/mL), free radicals were stained with DHE, and a Muse^®^ Cell Analyzer was used for analysis; *n* = 3–4. (**c**) Cells seeded on glass slides were treated as in (**a**), and 5 μM MitoSOX™ Red reagent was added in the dark for 1 h at 37 °C. A fluorescence microscope was used to photograph the distribution of free radicals in the cells; *n* = 4–7. The results are presented as the mean ± SD and were analyzed using one-way ANOVA followed by Dunnett’s post hoc test. ** *p* < 0.01, compared with the untreated group. ^#^ *p* < 0.05, ^##^ *p* < 0.01, compared with the TNF-α treatment group.

Excess ROS causes oxidative damage and changes kidney tissue. We examined whether TNF-α induced ROS formation in rat RMCs. Cellular and mitochondrial ROS were measured by H_2_DHFDA or DHE and MitoSOX™ Red staining, respectively. These fluorescent dyes were used to label H_2_O_2_ or ^•^O_2_^−^. The RNS level was indirectly measured by Griess reagent assay. As shown in Supplementary Figure S3, TNF-α induced ROS generation in a time-dependent manner. To clarify the antioxidant effect of these active molecules in RMCs, we quantified ROS generation. As shown in Figure 3a,b, AC had the best antioxidant capacity compared with AF and PD. Mitochondria are the main source of free radicals produced by the human body. Moreover, 98% of the oxygen in the respiration chain will generate energy in the cells, whereas about 2% will become free radicals. Therefore, we also observed that these active molecules abrogated the ROS generated from mitochondria (Supplementary Figure S3c). Among them, AC and Res had better activity compared with the remaining molecules.

### 3.4. Res and Its Derivatives Relieve TNF-α-Induced iNOS Expression and NO Production in RMCs

**Figure 4:**
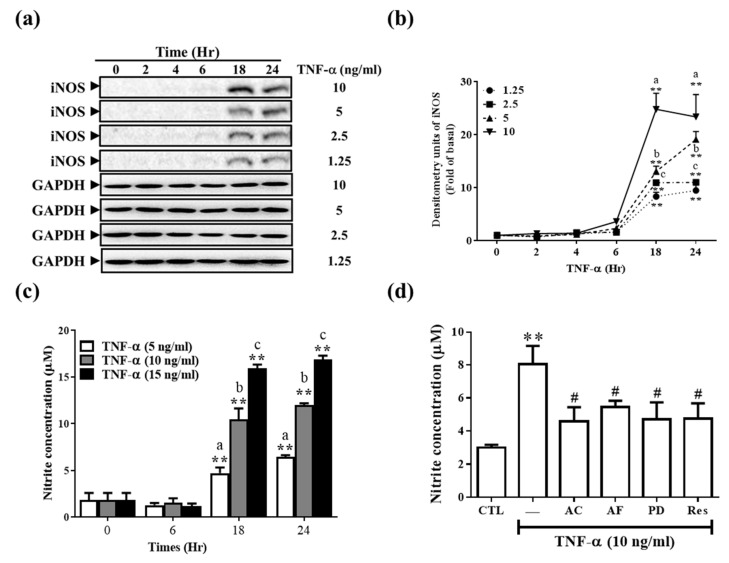
AC, AF, Res, and PD affected the TNF-α-induced iNOS expression and intracellular NO production. (**a**,**b**) TNF-α was used to stimulate RMCs at different concentrations and times. (**c**) Cells were treated with AC, AF, PD, and Res (10 μg/mL) for 2 h and then stimulated with TNF-α for 24 h. (**a**) The cell lysate was collected, and the expression of iNOS was analyzed by Western blotting using GAPDH as a loading control. (**b**) Quantitative graph for (**a**). (**c**,**d**) The supernatant was collected, and the NO content was indirectly analyzed using the Griess reagent analysis method. The results are presented as the mean ± SD from 3 experiments and analyzed (**b**,**c**) using two-way ANOVA followed by Tukey’s multiple comparisons post hoc test. *p* < 0.01; different letters represent significant differences between groups and (**d**) using one-way ANOVA followed by Dunnett’s post hoc test. ** *p* < 0.01, compared with the untreated group. ^#^ *p* < 0.05, compared with the TNF-α treatment group.

NO produced from the inflammatory mediator iNOS immediately reacts with free radicals (^•^O_2_^−^) to form RNS, thus damaging cells. RNS is considered critical in the pathogenesis and progression of CKD [4,5]. Next, we examined cellular iNOS expression in response to TNF-α. As shown in Figure 4a,b, TNF-α induced iNOS protein expression in a time- and dose-dependent manner. NO in oxygen-containing aqueous solutions has a short half-life, which is often attributed to its rapid oxidation to nitrite. Therefore, nitrite production indirectly indicates the NO level. Here, nitrite production (i.e., NO level) was similar to the iNOS expression pattern (Figure 4c). TNF-α stimulation at 18 and 24 h yielded significant differences between various concentrations, respectively (Figure 4c). Res and its derivatives significantly abrogated nitrite production (i.e., NO level) (Figure 4d).

### 3.5. TNF-α Induces MMP-9 and COX-2 Expression via the ROS Generated from Mitochondria and NADPH Oxidase in RMCs

**Figure 5:**
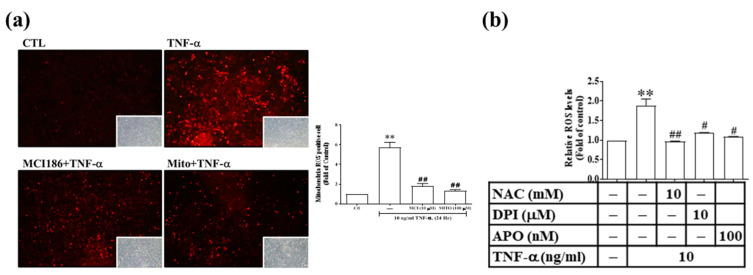

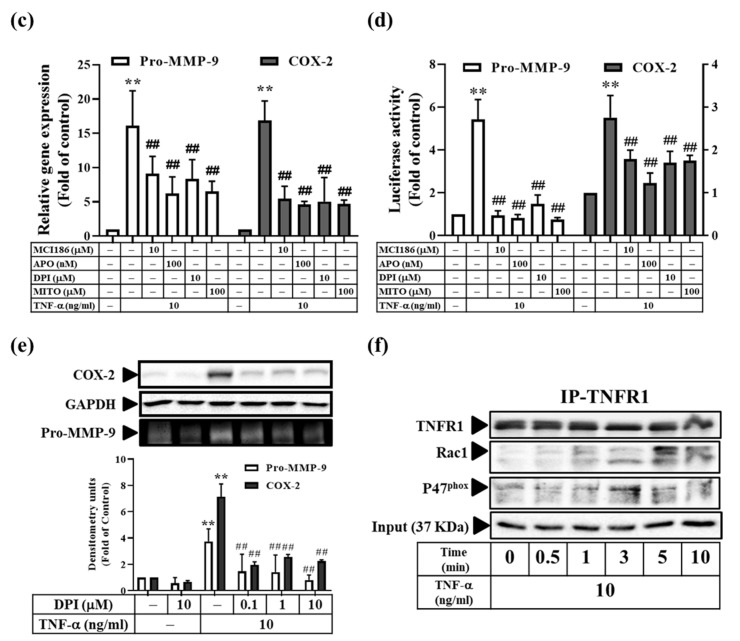
The ROS generated by the complex of TNFR1/TRAF2/Rac1/p47^phox^ participated in the TNF-α-induced upregulation of COX-2 and pro-MMP-9. (**a**) Cells seeded on glass slides were pre-treated with MCI186 and mito-TEMPO, followed by TNF-α treatment, then stained with MitoSOX™ Red reagent and photographed using a fluorescence microscope. (**b**) Cells were pre-treated with NAC, DPI, and APO, followed by TNF-α treatment. The cell lysate was collected, and the H_2_DCF fluorescence value was read. (**c**,**d**) Cells were pre-treated with MCI186, APO, DPI, and mito-TEMPO, followed by TNF-α treatment. (**c**) Real-time PCR was used to analyze the MMP-9 and COX-2 mRNA transcripts. (**d**) Luciferase assay was used to analyze the MMP-9 and COX-2 promoter activity. (**e**) Cells were pre-treated with various concentrations of DPI, followed by TNF-α treatment for 24 h (for COX-2) and 48 h (for MMP-9). MMP-9 enzyme activity was observed by gelatin zymography, and COX-2 expression was assessed by Western blotting. (**f**) The whole-cell lysate (input, 1 mg) was reacted with an anti-TNFR1 antibody. After the reaction, 50% protein A-agarose magnetic beads were mixed evenly at 4 °C for 1 day. The antigen–antibody conjugates were collected and prepared as the electrophoretic sample. Western blotting was used to analyze the binding of TNFR1, Rac1, or p47^phox^. The results are presented as the mean ± SD from 4–6 experiments and analyzed using one-way ANOVA followed by Dunnett’s post hoc test. ** *p* < 0.01, compared with the untreated group. ^#^ *p* < 0.05, ^##^ *p* < 0.01, compared with the TNF-α treatment group.

We also explored whether the ROS generated from NADPH oxidase and the mitochondrial respiratory chain system are involved in the expression of TNF-α-induced inflammatory proteins. MCI186 (which is a broad-based free radical inhibitor), apocynin (APO, a general ROS scavenger), diphenyleneiodonium chloride (DPI, a broad-spectrum flavoprotein inhibitor), and mito-TEMPO (a mitochondria-targeted free radical scavenger) were used. As shown in Figure 5a,b, MCI186, mito-TEMPO, NAC, DPI, and APO effectively alleviated TNF-α-induced oxidative stress. Next, to clarify whether ROS participate in TNF-α-induced MMP-9 and COX-2 expression, we performed pretreatment with MCI186, APO, DPI, and mito-TEMPO, followed by TNF-α. We found a significant reduction in the protein concentration, mRNA accumulation, and promoter activity of MMP-9 and COX-2 (Figure 5c–e). It has been reported that TNF-α induces the formation of the TNFR1/TRAF2/Rac1/p47^phox^ complex [26]. Therefore, we examined whether this phenomenon also occurs in TNF-α-challenged RMCs. After TNF-α stimulation for different periods, Rac1 began to bind to TNFR within 1–3 min, and the binding was most significant at 5 min. In addition, p47^phox^ binding to the complex of TNFR1/Rac1 occurred during 3–5 min (Figure 5f). These results indicate that the binding of TNF–α to TNFR1 recruits Rac1/p47^phox^ binding and immediately activates ROS production from NADPH, finally upregulating inflammatory proteins in RMCs.

### 3.6. TNF-α Induces Inflammatory Protein Expression via the c-Src-Dependent EGFR Transactivation Pathway in RMCs

**Figure 6:**
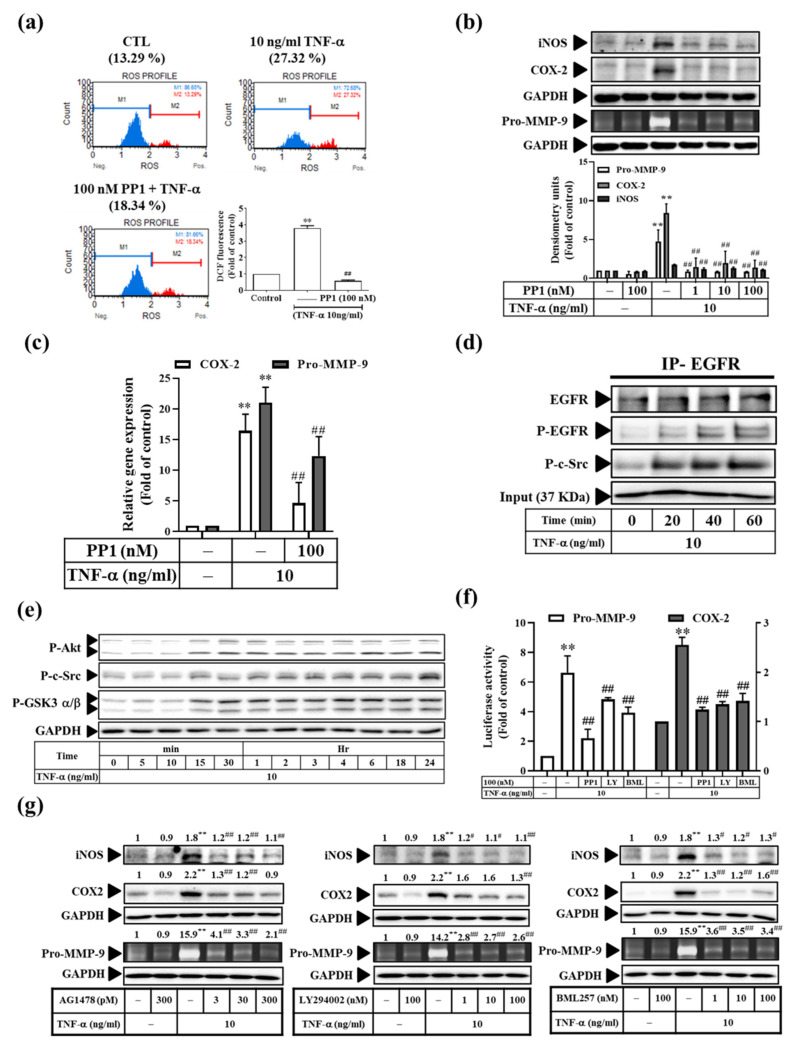
The c-Src/EGFR/PI3K/Akt/GSK3α/β pathway in TNF-α-induced COX-2 and pro-MMP-9 expression. (**a**) Cells were pre-treated with various concentrations of PP1, followed by TNF-α treatment for 6 h, collection of cells (1 × 10^6^ cells/mL), staining of free radicals with DHE, and analysis using a Muse^®^ Cell Analyzer. (**b**,**g**) Cells were pre-treated with various concentrations of (**b**) PP1 and (**g**) AG1478, LY294002, or BML257, followed by TNF-α treatment for 24 h (for COX-2 and iNOS) or 48 h (for MMP-9). MMP-9 enzyme activity was observed by gelatin zymography, and COX-2 and iNOS expression was detected by Western blotting. (**c**) Real-time PCR was used to analyze the MMP-9 and COX-2 mRNA transcripts. (**d**,**e**) After stimulation with TNF-α for various times, (**d**) the whole-cell lysate (input, 1 mg) was reacted with the anti-EGFR antibody. After co-immunoprecipitation, Western blotting was used with anti-EGFR, anti-P-EGFR, or anti-P-c-Src antibodies. (**e**) Western blotting was used to analyze the P-c-Src, P-Akt, and P-Gskα/β antibodies. (**f**) Cells were pre-treated with PP1, LY294002, and BML257, followed by TNF-α treatment. Luciferase assay was used to analyze the MMP-9 and COX-2 promoter activity. The results are presented as the mean ± SD from 4–6 experiments and analyzed using one-way ANOVA followed by Dunnett’s post hoc test. ** *p* < 0.01, compared with the untreated group. ^#^ *p* < 0.05, ^##^ *p* < 0.01, compared with the TNF-α treatment group.

It has been reported that the c-Src-dependent EGFR/PI3K/Akt pathway is involved in MMP-9 expression [27]; therefore, we examined whether the c-Src-dependent EGFR transactivation signaling pathway participates in the TNF-α-induced inflammatory protein expression in RMCs. First, pretreatment with PP1 (a c-Src inhibitor), AG1478 (an EGFR inhibitor), LY294002 (a PI3K inhibitor), or BML257 (an Akt inhibitor) was performed, followed by TNF-α stimulation. We found that these inhibitors reduced the TNF-α-induced levels of the iNOS, COX-2, and Pro-MMP-9 proteins (Figure 6b,g). Moreover, PP1, LY294002, or BML257 significantly attenuated the COX-2 and Pro-MMP-9 mRNA transcripts (Figure 6c) and promoter activity (Figure 6f). Next, we determined whether the c-Src and EGFR signaling pathway is activated in response to TNF-α. We found that TNF-α induced c-Src, Akt, and GSK3α/β phosphorylation (Figure 6e). This activation lasted from 15 m to 24 h. In addition, we observed that the phosphorylation of c-Src at 20 min led to its binding to EGFR, followed by the induction of its phosphorylation from 20 min to 60 min (Figure 6d). In addition, we investigated whether the TNF-α-regulated ROS generation is partially mediated via c-Src. Cell pretreatment with PP1 attenuated the TNF-α-induced ROS levels (Figure 6a). Taken together, these results indicate that the TNF-α-induced inflammatory protein expression occurs via the c-Src-dependent EGFR transactivation and NADPH oxidase pathways in RMCs.

### 3.7. TNF-α Induces Inflammatory Protein Expression via Three Types of MAPKs and NF-κB in RMCs

**Figure 7:**
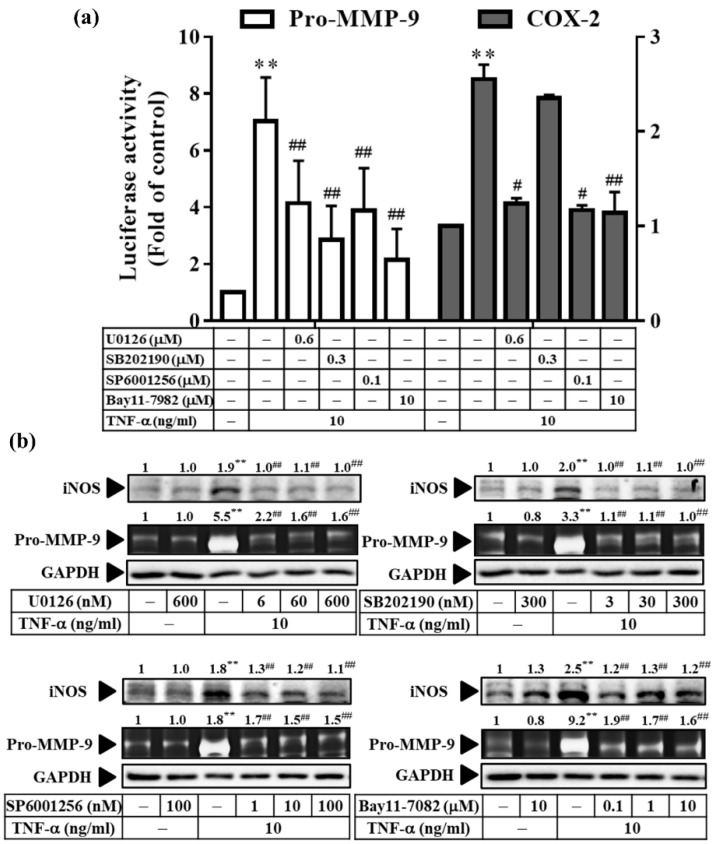
Involvement of MAPKs and NF-κB in TNF-α-induced, pro-MMP-9 expression. Cells were pre-treated with U0126, SB202190, SP600125, or Bay11-7082, followed by TNF-α treatment for 24 h (**a**) or 48 h (**b**). (**a**) Luciferase assay was used to analyze the MMP-9 and COX-2 promoter activity. (**b**) MMP-9 enzyme activity was observed by gelatin zymography, and Western blotting was used to analyze the expression of GAPDH (as a loading control). The results are presented as the mean ± SD from 4–6 experiments and analyzed using one-way ANOVA followed by Dunnett’s post hoc test. ** *p* < 0.01, compared with the untreated group. ^#^ *p* < 0.05, ^##^ *p* < 0.01, compared with the TNF-α treatment group.

Our previous study concluded that Res derivatives (AC, AF, and PD) inhibited cPLA2/COX-2/PGE2 expression via JNK1/2, ERK1/2, and NF-κB in RMCs [11]. Therefore, we investigated whether similar mechanisms were at play in TNF-α-induced MMP-9 and iNOS expression. As shown in Figure 7b, the inhibitors of three types of MAPKs (U1026, SB202190, and SP600125) significantly reduced the TNF-α-induced expression of the Pro-MMP-9 and iNOS proteins. In addition, this inhibition was also apparent in Pro-MMP-9 promoter activity. However, the effect on the COX-2 promoter activity was not exactly the same as that observed for the Pro-MMP-9 response in RMCs (Figure 7a).

### 3.8. TNF-α Induces Classical NF-κB Activation via ROS, Rather Than via the EGFR Pathway, in RMCs

**Figure 8:**
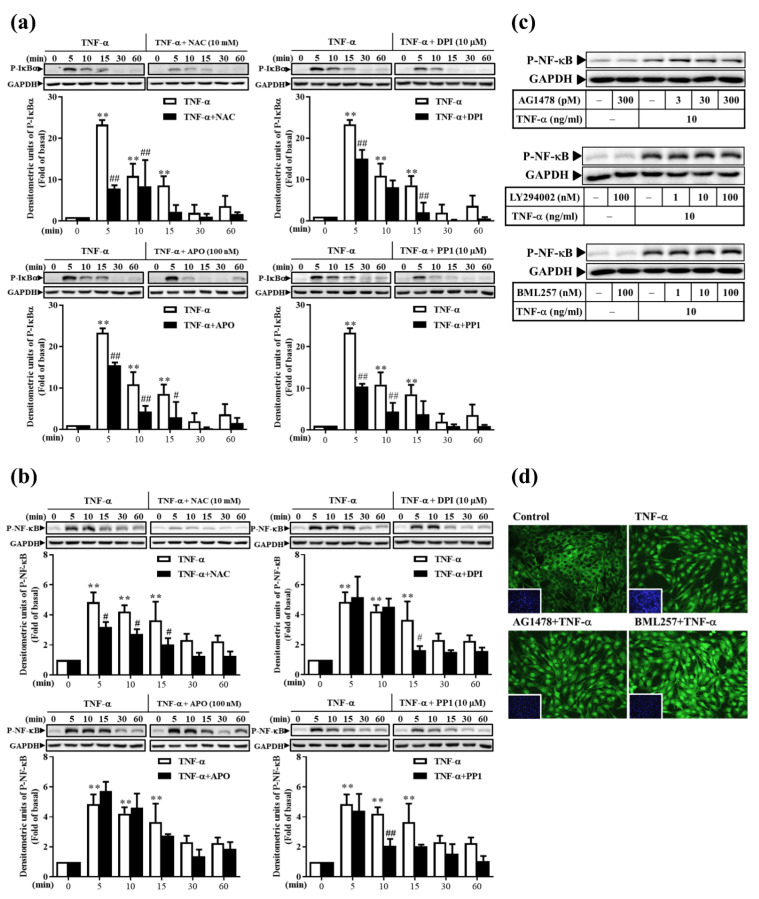
The TNF-α-induced NF-κB activation via c-Src-driven ROS was independent from the EGFR signaling pathway. (**a,b**) Cells were pre-treated with NAC, DPI, APO, or PP1, followed by TNF-α treatment for various times. (**a**) IκBα and (**b**) NF-κB activation was analyzed by Western blotting. (**c**,**d**) Cells were pre-treated with AG1478, LY294002, or BML257, followed by TNF-α treatment for 5 min. NF-κB activation was analyzed by (**c**) Western blotting and (**d**) immunofluorescence staining. The results are presented as the mean ± SD from 4–6 experiments and analyzed using one-way ANOVA followed by Dunnett’s post hoc test. **p < 0.01, compared with the untreated group. ^#^ *p* < 0.05, ^##^ *p* < 0.01, compared with the TNF-α treatment group.

Our previous study concluded that the activation of NF-κB induced by TNF-α is not related to MAPKs [11]. Therefore, we aimed to identify the upstream molecules that are involved in activating the induction of NF-κB by TNF-α. The pretreatment of cells with inhibitors related to the ROS and EGFR pathways was followed by a challenge with TNF-α. As shown in Figure 8a,b, NAC, DPI, APO, or PP1 attenuated TNF-α-induced IκBα and NF-κB phosphorylation at various time points. These inhibitory results were not observed after AG1478, LY294002, or BML257 treatment, as determined by Western blotting (Figure 8c) or immunofluorescence staining (Figure 8d). These findings indicate that the TNF-α-induced classical NF-κB activation occurs via ROS, rather than via the EGFR pathway, in RMCs.

## 4. Discussion

TNF-α is one of the important factors that cause kidney disease [28]. It can be secreted by RMCs, leading to the proliferation of the glomerular mesangial matrix, thus promoting the proliferation and differentiation of renal tubular epithelial and mesangial cells. Proliferation and glomerulosclerosis promote inflammation, directly or indirectly promoting the synthesis of the ECM. Studies have shown that in CKD induced by nephrectomy in rats, TNF-α was significantly upregulated in the blood and kidney tissues compared with the control group, and it was also related to the activation of NF-κB and the infiltration of macrophages [29]. Macrophages infiltrate the renal interstitium, and renal tubular epithelial cells and RMCs can produce large amounts of TNF-α. Therefore, in this study, an in vitro nephritis model was established using rat MCs, which were then subjected to TNF-α stimulation.

NF-κB is a key transcription factor for inflammatory cells [30]. When the cell is resting, p50/p65 exists in the cytoplasm and binds to IκBα to form a ternary body. When NF-κB is stimulated by extracellular signals, Ser^32^ and Ser^36^ of IκBα are phosphorylated, and p50/65 is released from the cytoplasm into the nucleus, thus regulating the corresponding downstream gene expression. NF-κB is directly involved in the occurrence of tubulointerstitial fibrosis. The activation of NF-κB triggers the accumulation of monocytes, leading to the inflammation of kidney tissues. In contrast, if NF-κB is inhibited, the incidences of renal tubular epithelial cell differentiation to myofibroblasts, ECM synthesis, fibrosis, and the degree of kidney damage are reduced. In organ fibrosis, NF-κB can promote the expression of genes encoding cytokines such as TNF-α, IL-8, and TGF-β, leading to fibrosis. At present, anti-fibrosis agents in different CKD disease models and the current state of drug trials and clinical development, mainly for the NF-κB pathway, have been reported [28]. Therefore, drugs that can target NF-κB to affect the expression of inflammatory proteins will help the treatment of CKD. Our previous study showed that the Res derivatives present in the root of VT also abolish the activity, nuclear translocation, and promoter activity of NF-κB challenged by TNF-α [11], suggesting a potential anti-inflammatory role for Res derivatives extracted from the root of VT for aiding the treatment of renal disease.

In the early stage of CKD, the activities of MMP-2 and MMP-9 are increased, which damages the kidney’s basement membrane, promotes the phenotypic transformation of renal tubular epithelial cells, and ultimately leads to the aggravation of ECM deposition. However, in the late stage of CKD, MMP-2 and MMP-9 are downregulated, resulting in insufficient ECM degradation; therefore, it is difficult to reverse fibrosis. The decrease of MMP-2 activity in the late stage of CKD is related to the increased endocytosis caused by hypoxia. However, the mechanism underlying the decrease in MMP-9 activity remains unclear [6]. Our data showed that the inflammatory MMP-9 protein was expressed in a time- and concentration-dependent manner after TNF-α stimulation, with the most significant manifestation observed at 18–24 h (Supplementary Figure S2). However, the expression of MMP-2 was not affected (Supplementary Figure S2a). These results of the stimulation of RMCs by TNF-α within 48 h are similar to the early stage of CKD, and MMP-9 plays an important role in this model.

ROS and RNS are considered critical in the pathogenesis and progression of CKD [4,5]. High glucose, H_2_O_2_, or AGE levels can induce ROS production in kidney cells, such as proximal tubular epithelial cells and mesangial cells [31]. High levels of ROS increase the expression and activity of TGFβ, leading to renal fibrosis through ECM accumulation and epithelial–to–mesenchymal transition [31]. ROS also reduce mitochondrial membrane permeability and regulate Bax/Bcl2, with its downstream caspase 3 activity leading to apoptosis [31]. In addition, ROS can also regulate the translocation of NF-κB from the cytoplasm to the nucleus by degrading phosphorylated IκBα and can activate the NLRP3 inflammasome pathway to express inflammatory cytokines and achieve an inflammatory response [31]. LPS induced the expression of iNOS and Nox4, followed by an increase in the levels of nitric oxide (NO) and ^•^O_2_^−^. NO then reacts with^•^O_2_^−^ to form ONOO^−^ (RNS), which causes mitochondrial dysfunction by interrupting mitochondrial oxidative phosphorylation and a further increase of ROS levels in mitochondria, therefore damaging human proximal tubular epithelial (HK-2) cells [32]. In the present study, cellular and mitochondrial ROS were measured by H_2_DHFDA or DHE and MitoSOX staining, respectively. The RNS level was indirectly measured by Griess reagent assay. As shown in Supplementary Figure S3a,b, the levels of H_2_O_2_ and ^•^O_2_^−^ in the cytosol increased in a time-dependent manner, and the maxima were observed at 6h. In addition, the ^•^O_2_^−^ level in mitochondria exhibited a similar trend, and a significant result was observed from 6 h up to 24 h (Supplementary Figure S3c). The nitrite level, which indirectly indicated NO production, showed an increased pattern in a time-dependent fashion (Figure 4c). NO then reacts with^•^O_2_^−^ to form ONOO^−^ (RNS), which causes mitochondrial dysfunction by interrupting mitochondrial oxidative phosphorylation and a further increase of ROS levels in mitochondria, therefore damaging RMCs. The intracellular ROS derived from mitochondria and NADPH oxidases activates downstream signaling molecules to regulate the expression of genes involved in the remodeling of the ECM in the diabetic kidney [33]. This prompted us to explore the ROS source in these cells. Our results revealed that mito-TEMPO treatment effectively alleviated TNF-α-induced mitochondrial ROS (mtROS) production (Figure 5a). In addition, TNF-α induced p47^phox^ binding to the complex of TNFR1/Rac1 (Figure 5f). Therefore, we speculated that ROS were derived from mitochondria and NADPH oxidase in RMCs. ROS derived from Nox4 contribute to many renal diseases [34,35,36,37]. Nox4 is constitutively activated and highly expressed in renal cells, including glomerular mesangial and podocyte cells. In kidney injury, Nox4 needs manganese-SOD (Mn-SOD) and copper/zinc-SOD (Cu/Zn-SOD) to catalyze the dismutation of ^•^O_2_^−^ to H_2_O_2_ in the mitochondrial matrix and intermembrane space, respectively [38,39]. In addition, NOX4-derived H_2_O_2_ and ^•^O_2_^−^ can be altered by coenzyme Q in TNF-α or LPS-induced endothelial inflammation [40]. Angiotensin II-activated Nox4 on the outer membrane in mitochondria generates mtROS and activates downstream protein kinase C (PKC) epsilon (PKCε). The mtROS causes the phosphorylation and opening of a mitochondrial adenosine triphosphate-sensitive potassium K channel, which decreases mitochondrial membrane potential depolarization. The activation of PKCε activates Nox2 through the p47^phox^ subunit, inducing ROS production [38]. The mtROS activates NF-κB by deactivating IKKγ in macrophages [41]. However, this mechanism has not been investigated in kidney diseases. Therefore, we speculated that TNF-α-induced mtROS generation might be partially through Nox4 activation in RMCs. In addition, TNF-α induced p47^phox^ binding to the complex of TNFR1/Rac1 (Figure 5f). Among NOXs in renal cells, Nox4 does not contain Rac binding sites, and Rac1 is ubiquitously expressed [42]. Moreover, ROS generation by Nox4 requires assembly with Nox2 or Nox1 [43,44,45]. Therefore, we speculated that Nox2 or Nox1 also partially participated in ROS generation in RMCs. In addition, among the Res derivatives at the same concentration, AC (a trimer of Res) exhibited the best antioxidant effect in RMCs (Figure 3). However, all Res derivatives inhibited TNF-α-induced NO levels (Figure 4d), thus possibly reducing RNS damage to cells. We also demonstrated that the interaction between the chemical structure of Res derivatives and DPPH^•^ free radicals underlay their ability to scavenge free radicals. Among the Res derivatives at the same concentration, PD (a Res glycoside) and Res had the best DPPH^•^ free radical scavenging activity (Figure 2a–d) within a short time. We speculate that this may be because of their small structure and ease of interaction with DPPH^•^ in space. Therefore, PD and Res may be best for scavenging intercellular ROS. In addition, the structure of Res derivatives is rich in hydroxyl groups, rendering it challenging for them to penetrate cells and perform ROS clearance. Previous studies have proposed that Res binds to the cytosolic estrogen receptors alpha and beta in MCF-7, rat uteri, and CHO-K1 cells [46]. The aryl hydrocarbon receptor located at the cell surface interacts with polydatin [47]. Ampelopsin exerts its anti-cancer ability through the regulation of growth factor receptor (VEGFR2 and PDGFRβ) and TRAIL/TRAIL-R pathways [48]; moreover, its anti-inflammatory ability is exerted through the modulation of the toll-like receptor 4 (TLR4) pathway [49] or binding to the ryanodine receptor [50]. All of these receptors have been reported to be expressed in HaCaT cells [51,52,53,54]. Therefore, we speculate that Res derivatives may downregulate intracellular ROS through binding to their receptors. However, there is currently no literature detailing the receptors for AC or AF binding or activity, and further confirmation is needed in future studies.

The root and stem of VT contain AC and Res, whereas the leaves do not. The VT root is rich in AC, and its content (about 3.57 mg/g) is much higher than that of Res (about 0.33 mg/g). Both AC and Res were detected in *V. vinifera* L., *V. kelungensis* Moriyama, and *Ampelopsis brevipedunculata* (Maxim.) Traut. Unlike Res, AC is not detected in *V. thunbergii* var. taiwaniana Lu. or *Cissus* *repens* Lam [55] in Taiwan. Many Res oligomers and polyphenol compounds are also present in the root of VT, all of which are stilbenes. The Res oligomers comprise 2–8 subunits of Res in their structure and have similar biological activities, including anti-cancer, anti-diabetic, anti-bacterial, and cardiovascular-protective effects [56], and improve psoriasiform lesions [9]. However, no literature has discussed the effect of VT on renal disease. In addition, the bioavailability and stability of Res are poor; thus, its clinical medicinal application is restricted. Recently, many studies have revealed that Res derivatives are more effective in inhibiting tumor cell proliferation, inflammation, and antifungal activity compared with Res [19,20,21]. Our previous study revealed that the products purified from VT (AC and AF), Res, and PD can inhibit the expression of cPLA2/COX-2/PGE2 induced by TNF-α [11]. Consistent with previous findings, in this study, treatment with Res derivatives effectively attenuated TNF-α-induced inflammatory mediators, e.g., MMP-9 expression at the mRNA and protein levels (Figure 1). In addition, among the Res derivatives at the same concentration, AC (a trimer of Res) exhibited the best anti-inflammatory effect in RMCs (Figure 1a). We propose that AC might be superior to Res for GN treatment based on its observed antioxidant and anti-inflammatory effects.

Next, we explored the molecular mechanisms underlying these effects. Our results revealed that the anti-inflammatory effect of Res derivatives occurred via the inhibition of ROS derived from mitochondria (Figure 5a,c–e) and NADPH oxidase (Figure 5b–e); attenuation of RNS derived from iNOS (Figure 4); and reduction of the activation of ERK1/2, JNK1/2, and NF-κB [11]. In addition, SB202190 inhibited the TNF-α-induced MMP-9 and iNOS expression at the protein and promoter activation levels (Figure 7). This result indicates that p38 participates in TNF-α-induced MMP-9 and iNOS expression, but not in that of COX-2, which is consistent with the findings of our previous study [11]. In addition, TNF-α directly induced the expression of inflammatory proteins (e.g., COX-2, MMP-9, or iNOS) at the protein (Figure 6b) and mRNA (Figure 6c) levels via the association of TNFR with Rac1 and p47^phox^ (Figure 5f), thus activating the NAPDH oxidase-dependent MAPK and NF-κB pathways (Figure 8a,b), and via the alternative transactivation of the c-Src-dependent EGFR/PI3K/Akt pathway (Figure 6). c-Src not only participated in ROS production (Figure 6a), but also bound to EGFR, leading to EGFR autophosphorylation (Figure 6d) and downstream Akt and GSKα/β activation (Figure 6e), which was consistent with a previous study [57]. Arsenic trioxide-stimulated EGFR transactivation was mediated through the NADPH oxidase/c-Src pathway in human keratinocytes [57]. However, the EGFR/PI3K/Akt pathway did not affect NF-κB activation and nuclear translocation (Figure 8c,d). Previous studies have proposed that Res resists the angiotensin II-induced hypertrophy of vascular smooth muscle cells via NADPH oxidase, c-Src, EGFR, and PDGFR activation and associates with the ERK1/2 and Akt signaling pathways [58]. Therefore, understanding their underlying anti-inflammatory mechanisms will promote the development of the Res derivatives in the root of VT in Taiwan for pharmaceutical applications.

## 5. Conclusions

**Figure 9:**
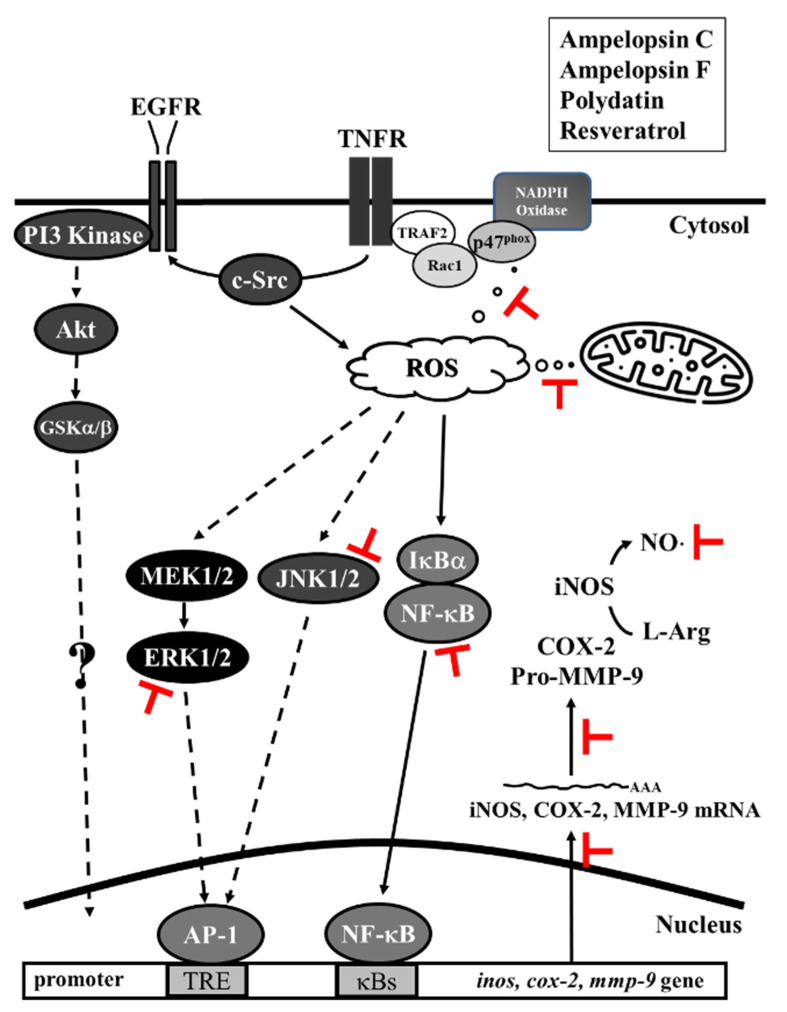
Pharmacological diagram of the inhibition of TNF-α-induced inflammatory proteins by Res derivatives. The TNF-α-induced activation of MAPKs and NF-κB was mediated by ROS derived from mitochondria and NADPH oxidase. TNF-α binding to TNFR recruited Rac1 and p47^phox^, thus activating NAPDH oxidase. In addition, the expression of MMP-9 induced by TNF-α was mediated by the MAPK, EGFR, and classical NF-κB pathways, respectively. However, Res derivatives inhibited the ROS derived from mitochondria and NADPH oxidase, the RNS derived from iNOS, the activation of ERK1/2 and JNK1/2, and NF-κB, which were induced by TNF-α.

We demonstrate that TNF-α directly induced the expression of inflammatory proteins via the association between the TNFR and Rac1 and p47^phox^ via the activation of the NAPDH oxidase-dependent MAPK and NF-κB pathways or via the alternative transactivation of the c-Src-dependent EGFR/PI3K/Akt pathway. The anti-inflammatory effects of the Res derivatives were exerted via the inhibition of the ROS derived from mitochondria and NADPH oxidase and the RNS derived from iNOS as well as the activation of the ERK1/2, JNK1/2, and NF-κB pathways (Figure 9). Overall, the results of this study promote the understanding of the various activities of Res derivatives from VT root extracts in Taiwan and their pharmacological mechanisms.

## Data Availability

Data is contained within the article and supplementary material.

## Supplementary Materials

The following supporting information can be downloaded at: https://www.mdpi.com/article/10.3390/antiox11050835/s1, Supplementary Figure S1: The effect of AC, AF, PD or Res on cell viability; Supplementary Figure S2: TNF-α induced pro-MMP-9 protein expression in a time- and dose-dependent manner; Supplementary Figure S3: TNF-α induced ROS generation in a time-dependent manner.
