# Supplementary material for: Anti-Inflammatory Effect of Resveratrol Derivatives via the Downregulation of Oxidative-Stress-Dependent and c-Src Transactivation EGFR Pathways on Rat Mesangial Cells
Source: Antioxidants (Basel). 2022 Apr 25;11(5):835. doi: 10.3390/antiox11050835 (PMC9138040; doi:10.3390/antiox11050835)
Supplement: Supplementary file 1 [file antioxidants-11-00835-s001.zip › antioxidants-1669217-supplementary.pdf]

Article

Anti-Inflammatory Effect of Resveratrol Derivatives via the Downregulation of Oxidative-Stress-Dependent and c-Src Transactivation EGFR Pathways on Rat Mesangial Cells

I-Ta Lee [1,†], Horng-Chyuan Lin [2,†], Tse-Hung Huang [3,4,5], Chi-Nan Tseng [6,7,8], Hao-Tsa Cheng [8,9,10], Wen-Chung Huang [3,8,11,12] and Ching-Yi Cheng [3,13,*]

1 School of Dentistry, College of Oral Medicine, Taipei Medical University, Taipei 11031, Taiwan; itlee0128@tmu.edu.tw
2 Department of Thoracic Medicine, Chang Gung Memorial Hospital at Linkou, Taoyuan 33305, Taiwan; College of Medicine, Chang Gung University, Taoyuan 33302, Taiwan; lhc53424@cgmh.org.tw
3 Graduate Institute of Health Industry Technology, Research Center for Chinese Herbal Medicine and Research Center for Food and Cosmetic Safety, Chang Gung University of Science and Technology, Taoyuan 33303, Taiwan; kchuang@cgmh.org.tw (T.-H.H.); wchuang@mail.cgust.edu.tw (W.-C.H.)
4 Department of Traditional Chinese Medicine, Chang Gung Memorial Hospital, Keelung 20401, Taiwan
5 School of Nursing, National Taipei University of Nursing and Health Sciences, Taipei 11219, Taiwan
6 Division of Cardiac Surgery, Department of Thoracic and Cardiovascular Surgery, Chang Gung Memorial Hospital at Linkou, Taoyuan 33305, Taiwan; chinan.tseng@cgmh.org.tw
7 Department of Molecular Medicine and Surgery, Karolinska Institute, 17177 Stockholm, Sweden
8 Department of Medicine, Chang Gung University, Taoyuan 33302, Taiwan; hautai@adm.cgmh.org.tw
9 Department of Gastroenterology and Hepatology, Chang Gung Memorial Hospital at Linkou, Taoyuan 33305, Taiwan
10 Graduate Institute of Clinical Medicine, College of Medicine, Chang Gung University, Taoyuan 33302, Taiwan
11 Division of Allergy, Asthma and Rheumatology, Department of Pediatrics, Chang Gung Memorial Hospital at Linkou, Taoyuan 33305, Taiwan
12 Department of Pediatrics, New Taipei Municipal TuCheng Hospital, New Taipei 23652, Taiwan; Chang Gung Memorial Hospital at Linkou, Taoyuan 33305, Taiwan
13 Department of Pulmonary Infection and Immunology, Chang Gung Memorial Hospital at Linkou, Taoyuan 33305, Taiwan
* Correspondence: jennycheng@mail.cgust.edu.tw; Tel./Fax: +886-3-211-8866
† These authors contributed equally to this work.

Citation: Lee, I.-T.; Lin, H.-C.; Huang, T.-H.; Tseng, C.-N.; Cheng, H.-T.; Huang, W.-C.; Cheng, C.-Y. Anti-Inflammatory Effect of Resveratrol Derivatives via the Downregulation of Oxidative-Stress-Dependent and c-Src Transactivation EGFR Pathways on Rat Mesangial Cells. *Antioxidants* **2022**, *11*, 835. https://doi.org/10.3390/antiox11050835

Academic Editors: Maria Letizia Manca, Amparo Nacher, Mohamad Allaw, Matteo Perra and Ines Castangia

Received: 22 March 2022
Accepted: 22 April 2022
Published: 25 April 2022

Publisher's Note: MDPI stays neutral with regard to jurisdictional claims in published maps and institutional affiliations.

Copyright: © 2022 by the authors. Submitted for possible open access publication under the terms and conditions of the Creative Commons Attribution (CC BY) license (https://creativecommons.org/licenses/by/4.0/).

Supplementary Information

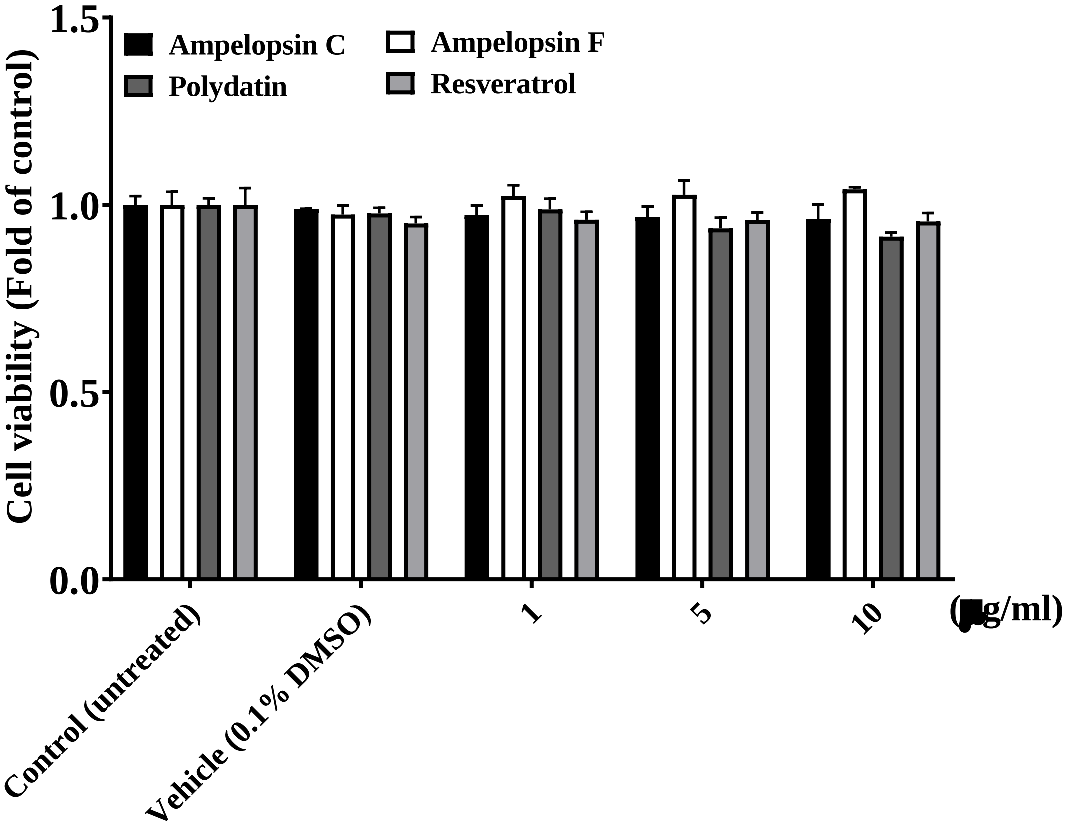

Supplementary Figure S1. The effect of AC, AF, PD or Res on cell viability. Cells were treated with various concentrations of AC, AF, PD or Res for 24 h. Cell viability was assayed using the MTT assay. Data are expressed as the mean ± SD from 3 experiments and analyzed using one-way ANOVA followed by Dunnett's post hoc test.

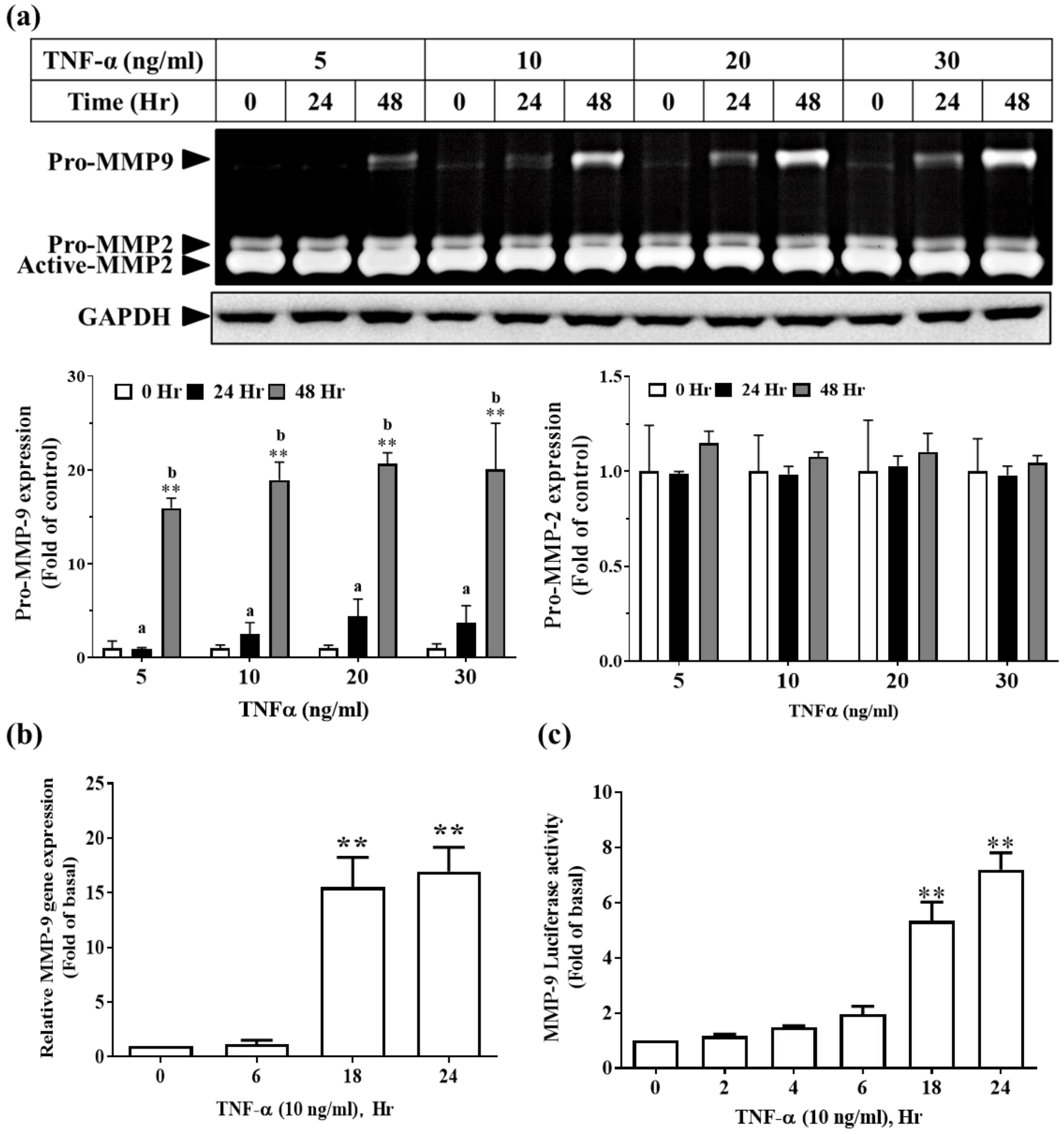

Supplementary Figure S2. TNF-α induced pro-MMP-9 protein expression in a time- and dose-dependent manner. TNF-α stimulated RMCs at different concentrations and times. (**a**) MMP-9 enzyme activity was determined by gelatin zymography, and Western blotting analyses were used to determine the expression of GAPDH (as a loading control). (**b**) The *MMP-9* mRNA transcripts were determined by real-time PCR. (**c**) Cells were transformed with the *MMP-9* promoter-luciferase plasmids by electroporation. After TNF-α treatment, a luciferase assay was used to analyze the promoter activity of *MMP-9*. The results are presented as the mean ± standard deviation (SD) from 4 – 6 experiments and analyzed (**a**) using two-way ANOVA followed by Tukey's multiple comparisons post hoc test. $P < 0.01$, different letters represent significant differences between groups; (**b**, **c**) using one-way ANOVA followed by Dunnett's post hoc test. ** $P < 0.01$, compared with the untreated group.

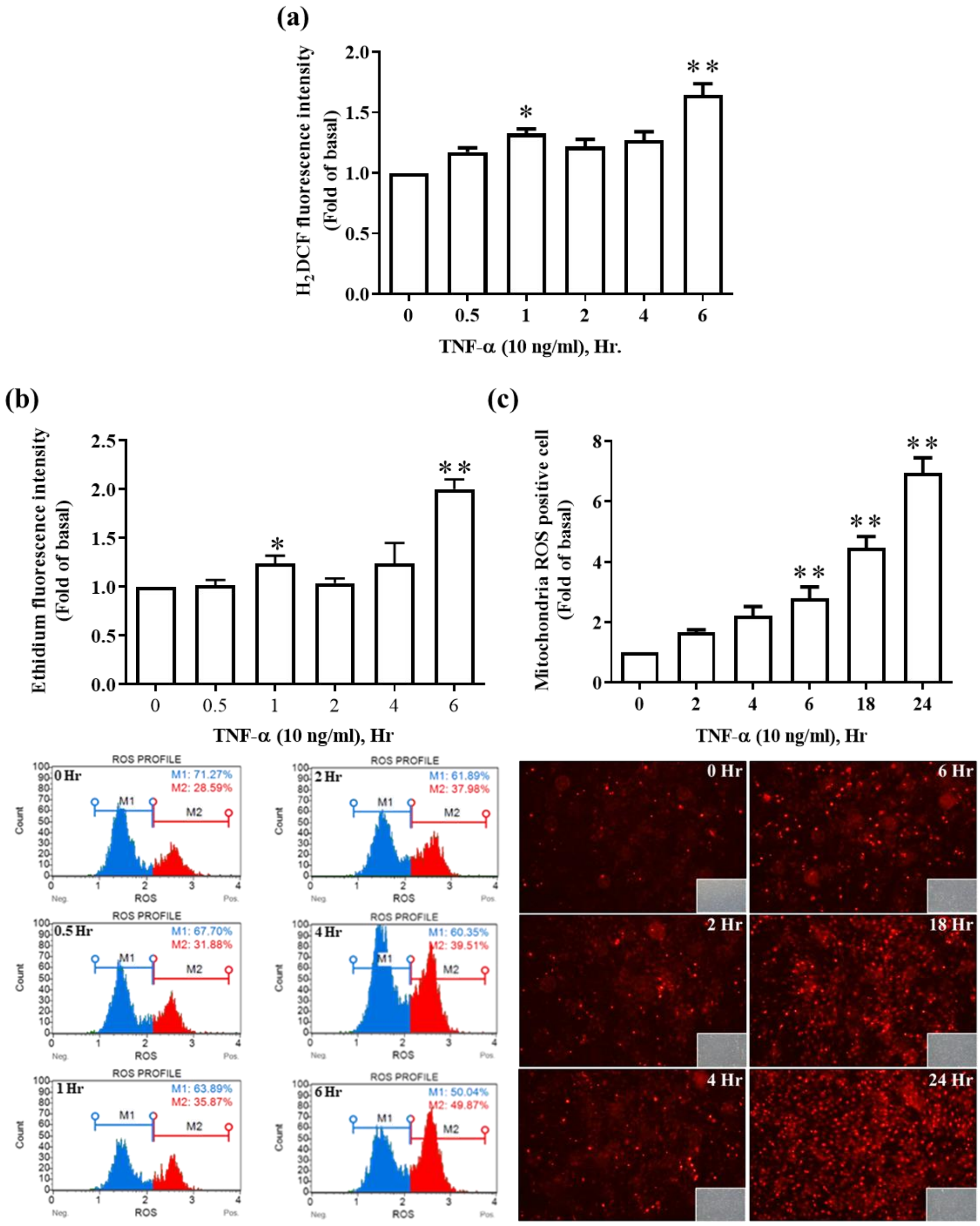

Supplementary Figure S3. TNF-α induced ROS generation in a time-dependent manner. (**a**) H_2DCFDA (4 μM) was added to starved cells at 37°C for 30 min and stimulated for different times with TNF-α (10 ng/ml). Cell lysates were collected and the H_2DCF fluorescence values were read on a SpectraMax i3 microplate reader (excitation at 485 nm, emission detection at 530 nm); n = 4 – 8. (**b**) After the same treatment as that described in (**a**), cells were harvested (1 × 10^6 cells/ml), free radicals were stained with DHE, and a Muse® Cell Analyzer was used for analysis; n = 3 – 4. (**c**) Cells seeded on glass slides were treated as in (**a**), and 5 μM MitoSOX™ Red reagent was added in the dark for 1 h at 37°C. A fluorescence microscope was used to photograph the distribution of free radicals in the cells; n = 4 – 7. The results are presented as the mean ± SD and analyzed using one-way ANOVA followed by Dunnett's post hoc test.

* $P < 0.05$, ** $P < 0.01$, compared with the untreated group.
